# Cul5: immune cell function and therapeutic potential

**DOI:** 10.3389/fimmu.2026.1808586

**Published:** 2026-04-15

**Authors:** Siera A. Tomishima, Paula M. Oliver

**Affiliations:** 1 Department of Pathology and Laboratory Medicine, Perelman School of Medicine, University of Pennsylvania, Philadelphia, PA, United States; 2 Division of Protective Immunity, Children’s Hospital of Philadelphia, Philadelphia, PA, United States

**Keywords:** cullin 5, E3 ubiqitin ligase, immune cells, SOCS proteins, ubiquitin proteasomal system

## Abstract

A primary function of immune cells is to protect against pathogens. To do this, cells surveil the body using receptors on their surface that can detect antigens from the invading organism or sense cytokines that act as danger signals. These receptors activate transcriptional programs that allow the cells to mount a response appropriate for the pathogen detected. To rapidly switch into an activated state, or to return to homeostasis, immune cells must initiate and terminate signaling pathways. Immune cells use post-transcriptional regulatory processes as one means to quickly change cellular behavior. This can be mediated by kinases and phosphatases that turn signaling pathways on or off. An additional important mechanism for downregulating immune effector cells is mediated by E3 ubiquitin ligases (E3s), which promote the degradation of receptors and their downstream signaling mediators. Ubiquitin ligases are enzymes that add ubiquitin modifications to specific protein substrates, targeting them for degradation via recruitment to the proteasome or altering their localization and activity. Cullin 5 (Cul5) is a scaffold protein that forms a multiprotein complex called Cullin Ring Ligase 5 (CRL5). To select substrates, CRL5 engages with Suppressors of Cytokine Signaling (SOCS)-box containing proteins. Collaborating with different SOCS-box containing substrate receptors allows Cul5 to promote selected protein degradation in a cell type- and context-specific manner. CRL5 and SOCS-box containing proteins regulate cytokine signaling to control proliferation, differentiation and immune functions in various cell types. Here, we give an overview of the ubiquitin proteasome system (UPS) and review new insights that advance our understanding of how Cul5 and CRL5 complexes regulate immune cell function. We then discuss ongoing therapeutic strategies that target various components of the UPS, and highlight the potential for new therapies targeting CRL5 for a range of diseases.

## Introduction

### Ubiquitin proteasome system

Ubiquitination is a post-translational modification that can impact various aspects of protein function, including half-life, localization, and protein complex formation. The addition of monomers or ubiquitin chains to substrates is a multi-step process that requires at least 3 types of ubiquitin pathways enzymes. The E1 ubiquitin activating enzyme (E1) transfers ubiquitin to an E2 ubiquitin conjugating enzyme (E2). E2s can then transfer ubiquitin to a catalytically active E3 ubiquitin ligase (E3) – including HECT and RBR E3s – or directly to the substate of a non-catalytic RING E3. After the initial ubiquitin has been added, ubiquitin chains can be formed on the substrate by the addition of ubiquitin to lysine residues on ubiquitin. There are 7 lysines (K) on ubiquitin that can be used to form chains: K6, K11, K27, K33, K48 and K63. Additionally, linear ubiquitin chains can be formed using the N-terminal methionine. Polyubiquitin chains formed on lysines can be homotypic, heterotypic, or branched. Homotypic chains are formed by the addition of ubiquitins sequentially to the same residue while heterotypic chains have ubiquitin added to different lysine residues. Branched chains have ubiquitin modifications that form in multiple directions from the original chain. These various ubiquitin chain types, or codes, have unique roles in directing protein function (1, 2). E3s are highly conserved across eukaryotes and are required for many facets of development and cellular function. The human genome encodes two E1s, between 30–50 E2s, and over 600 E3s, each of which targets multiple protein substrates. Mutations in E3s are linked with a myriad of diseases, from multiple sclerosis to Parkinson’s (3). One of the largest families of ligases are Cullin RING Ligases (CRLs).

### Cullins and cullin RING ligases

There are eight Cullin proteins in humans: Cul1, Cul2, Cul3, Cul4a, Cul4b, Cul5, Cul7, and Cul9. Cullins are scaffold proteins that facilitates the assembly of an E3 ubiquitin ligase (E3) called CRL. Each Cullin has a unique structure and therefore recruits distinct binding partners to form their CRL which contains an adaptor protein, a catalytic subunit and an adaptor. There are two catalytic subunits that Cullins use to recruit E2s to facilitate ubiquitination. Cul1, 2, 3, 4a and 4b use a catalytic subunit called RING-Box 1 (Rbx1), while Cul5 preferentially uses RING-Box 2 (Rbx2). Cul1 and Cul7 use Skp1 as an adaptor to recruit F-box proteins that help control cell cycle progression. Cul2 recruits VHL-box proteins and plays a crucial role in hypoxic response. Cul5 recruits SOCS-box proteins which are critical regulators of cytokine signaling. Cul3 partners with BTB domain proteins such as KLHL and SPOP, regulating cell cycle transitions. Finally, Cul4a and Cul4b use DDB1 to bind DCAH/H-box proteins, mediating DNA replication and damage repair (4, 5). CRLs have diverse functions in regulating biological processes and control the degradation of about 20% of all cellular proteins (6).

Cullin family proteins have been implicated in various human diseases. Dysregulated expression of all eight human Cullins have been associated with several cancers, and are often linked to worse prognosis, decreased survival and increased metastasis (7, 8). Variants in the Cul1 gene were identified in patients with neurodevelopmental disorders and zebrafish knockout studies demonstrated its essential function in central nervous system development (9). Mutations of Cul3 can cause neurodevelopmental disorders as well as a kidney disorder called Familial hyperkalemic hypertension (10, 11). X-linked mental retardation is caused by mutations in Cul4b, resulting from deficiencies in neuronal development and synaptic function (12, 13). Mutations in Cul7 cause 3-M syndrome, a condition caused by pre- and postnatal growth retardation, likely due to accumulation of insulin receptor substrate-1 and dysregulation of insulin-like growth hormone (14, 15). In a recent review on the link between CRL substrate receptors and rare diseases, 93 of the putative 267 substrate receptors utilized by Cullins were linked to germline disorders (16). It is likely that as genome-scale sequencing becomes more widely performed we will identify more connections between CRL networks, their function in human biology, and how their perturbation leads to disease.

For this review, we will focus on the function of CRL5 in immune cell regulation, its known roles in human disease, and discuss how it may be targeted for immune or cancer therapies.

### Cul5 and CRL5 complexes

Cullin 5 (Cul5) is a scaffold protein that facilitates the assembly of an E3 ubiquitin ligase (E3) called CRL5. At the N-terminus, Cul5 simultaneously binds two adaptors, Elongins B and C. These adaptors recruit a SOCS-box containing substrate receptor that is associated with its target substrate. The Cul5 C-terminus binds Rbx2, which recruits an E2. Once the complex has assembled, the E2 ubiquitinates the substrate (17). There are over 35 proteins in the mammalian genome that contain a SOCS-box motif, raising the possibility Cul5 has 35 potential substrate receptors. However, SOCS-box motifs have varying affinities for Cul5, supporting that Cul5 preferentially binds specific SOCS-box containing proteins. It can be difficult to study Cul5 association with substrate receptors *in vivo*, due to the transient nature of these interactions. Thus, many studies have relied on overexpression and *in vitro* systems.

CRL5 can form a complex with multiple types of substrate receptors *in vitro* including suppressor of cytokine signaling (SOCS) proteins, Ankyrin and SOCS box (ASB)-containing proteins, WD repeat-containing SOCS-box (WSB) proteins and SPRY-containing SOCS-box (SPSB) proteins. SOCS proteins, unlike the other families of SOCS-box containing proteins, have an SH2 domain that allows them to bind phosphorylated tyrosines on target substrates. SOCS1 and SOCS3 also contain a Kinase Inhibitory Region (KIR) that allows them to directly block the active site of kinases, preventing their ability to phosphorylate downstream pathway intermediates (18). The extent to which SOCS proteins require Cul5 for their function remains unclear. In fact, studies in which the SOCS-box of SOCS1 and SOCS3 were deleted support that some of their functions are Cul5 independent (19, 20). With that said, Cul5 substrate receptors can recruit one or more protein targets for ubiquitination, resulting in their proteasomal degradation (21). Degradation of these protein targets can impact signaling pathways in immune cells (22). Cul5 degrades substrates responsible for modulation of cell cycle, survival, growth, proliferation, differentiation and cytokine signaling in multiple cell types (23–26).

Activation of the CRL5 complex requires another post-translational modification called neddylation, which involves the addition of Nedd8, to Cul5. The Nedd8 pathway shares similarities with the ubiquitination pathway, involving the stepwise transfer of Nedd8 from a NEDD8-Activating Enzyme (NAE) to a NEDD8-Conjugating Enzyme (UBE2M or UBE2F) which transfers Nedd8 to a Cullin. The neddylation of Cul5 changes the conformation of the CRL5 complex, allowing it to transfer ubiquitin to its substrates (27). The CRL5 complex can also be further regulated by interaction with other proteins that remove Nedd8. The COP9 Signalosome (CSN) binds neddylated Cul5 and promotes the removal of Nedd8 from the CRL5 complex (28). This allows for the binding of Cullin-Associated Nedd8-Dissociated Protein 1 (CAND1), which promotes the exchange of substrate receptors bound to unneddylated Cullin complexes. This is thought to facilitate binding of the complex to less abundant substrate receptors (29).

## CRL5 and SOCS proteins in immune cell function

SOCS proteins act as negative feedback rheostats to prevent excessive cytokine receptor signaling (30, 31). They can work alone, using their SH2 and/or KIR domains, or act as substrate receptors within a CRL5 complex to degrade signaling intermediates and restrict proliferation, differentiation and function of immune cells. One of the most well-studied roles of SOCS proteins is their function in providing negative feedback of JAK/STAT signaling. SOCS proteins can regulate JAK/STAT proteins in several ways: 1) limiting activity of JAKs through their kinase inhibitory region (SOCS1 and 3) (32, 33); 2) by working with Cullin 5 (Cul5) to ubiquitinate cytokine receptors, JAKs, or STATs (34); 3) competitive binding of STAT docking site on cytokine receptors (35); or by 4) steric hinderance of STAT dimers to prevent nuclear translocation (36).

**Figure 1:**
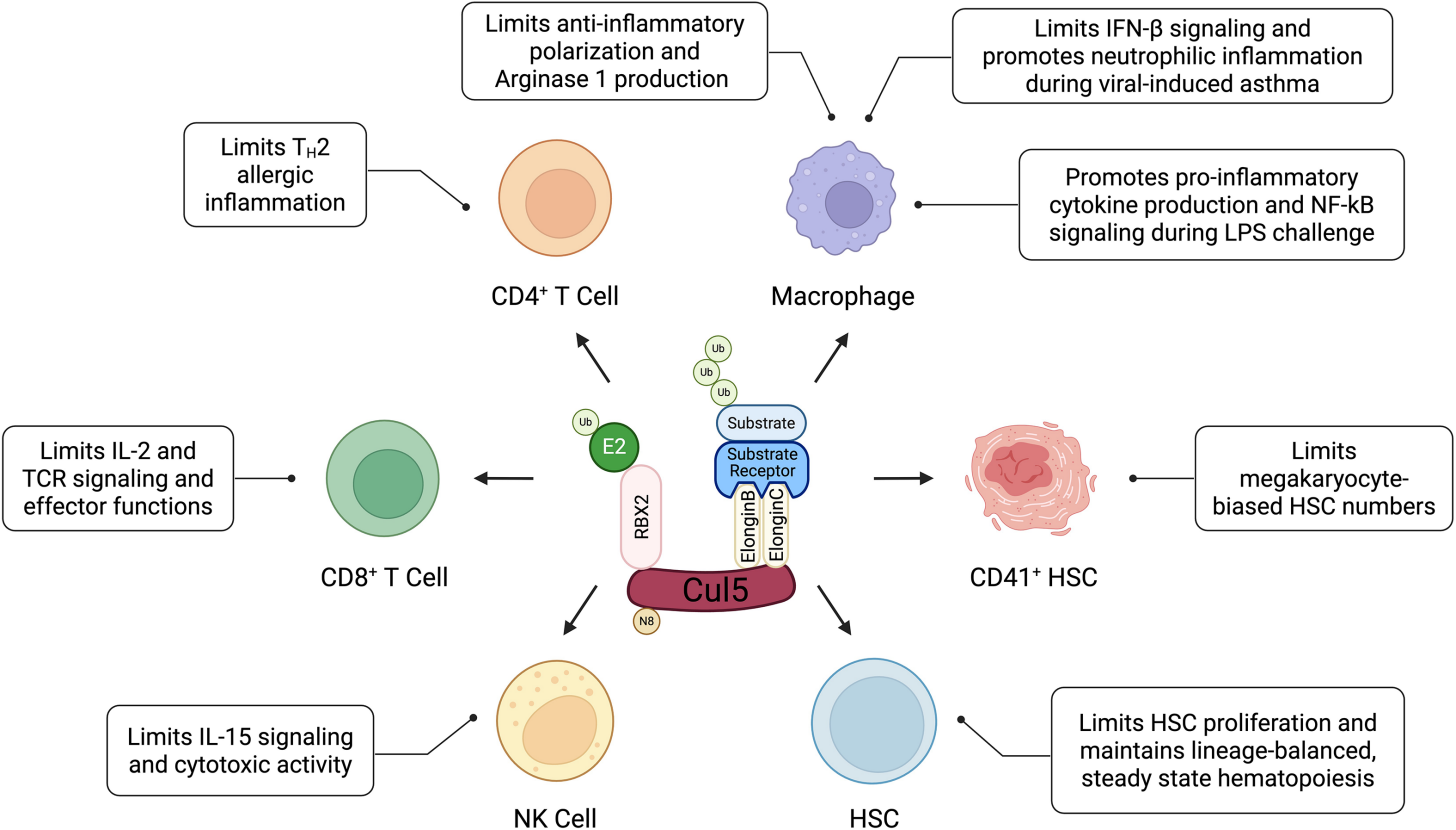
The modulatory role of Cul5 or CRL5 on immune cell functions including proliferation, activation, cytokine production, receptor signaling and differentiation/polarization.

Modulation of JAK/STAT signaling by SOCS proteins acting within a CRL5 complex allows cells to rapidly respond and adapt to environmental cues to maintain homeostasis or expand and differentiate during stress or infection. There are eight SOCS proteins – CISH, SOCS1, SOCS2, SOCS3, SOCS4, SOCS5, SOCS6, SOCS7 – which have roles in multiple immune cell types. CIS, SOCS1, SOCS2 and SOCS3 have been studied extensively for their role in regulating cytokine signaling in immune cells (37). As mentioned above, SOCS proteins do not always require Cul5 for their function, and vice versa. Since many studies on SOCS proteins have not directly interrogated their interactions with Cul5, it remains unclear in which cell types and scenarios they require each other. In the next few sections we will describe the research on SOCS proteins in innate (**Table 1**) and adaptive immunity (**Table 2**), as well as studies specifically on Cul5 in immune cell function (**Table 3**; **Figure 1**). In an effort to support clinical relevance, we will focus on findings of SOCS proteins and Cul5 from studies using mouse models and primary human samples.

**Table 1:** SOCS function and targets in innate immune cells.

SOCS protein	Immune cell type	Cytokine/ receptor	Affected pathway	Function	Ref
CIS	NK Cells	IL-15	JAK1	Limits proliferation, survival, IFNγ production and cytotoxic activity	(38)
	Dendritic Cells			Inhibits proliferation of DC progenitors to promote type 1 development	(39)
	Alveolar Macrophages		GATA2	Limits generation of foamy alveolar macrophages	(40)
SOCS1	Macrophages	TLR	NF-κB; STAT1	Prevent excessive immune activation in response to endotoxins	(41)
		IFN-β	STAT1	Inhibits CD40 expression	(42)
				Mediates pro- vs anti-inflammatory polarization	(43)
	Dendritic Cells	IL-12		Maintenance of self-tolerance	(44)
		IL-4 and IFNγ		Limits systemic autoimmunity	(45)
SOCS2	Dendritic Cells	TLR	STAT3	Limits IL-1β and IL-10 secretion	(46)
	Macrophages		NF-κB	Limits inflammasome signaling to mediate apoptosis	(47)
	NK Cells	IL-15	JAK2	Limits NK development	(48)
SOCS3	Macrophages	IL-6	STAT3		(49)
			NF-κB	Promotes pro-inflammatory polarization	(50)
	Neutrophils	G-CSF			(51, 52)

**Table 2:** SOCS function and targets in adaptive immune cells.

SOCS protein	Immune cell type	Cytokine/ receptor	Affected pathway	Function	Ref
CIS	CD8^+^ T Cells	TCR	PLC-γ1	Decreases proliferation and cytokine production	(53)
	CD4^+^ T Cells	TCR	MAPK	Mediates proliferation and survival	(54)
SOCS1	CD4^+^ T Cells	IL-6	STAT1	Limits T_H_1 differentiation	(55)
	CD4^+^ T Cells			Limits T_H_2 differentiation and IL-4 production	(56)
	T Cells	IL-7 and IL15		Mediates early T cell development	(57–60)
	Regulatory T Cells			Maintains Foxp3 expression by limiting IFNγ and IL-17 production	(61)
	Dendritic Cells		STAT3	Limits TGF-β production and T_reg_ differentiation and expansion	(62)
SOCS3	Natural Killer T Cells			Limits IL-4 and IFNγ production	(63)
	CD4^+^ T Cells			Promotes T_H_2 differentiation; limits T_H_1 and T_H_17 differentiation	(64–67)
	CD4^+^ T Cells	IL-2		Limits proliferation	(68, 69)
	CD8^+^ T Cells	IL-27		Limits proliferation	(68, 69)
	Plasma Cells	IL-6	STAT3	Mediates germinal center formation and function	(70)

**Table 3:** Cul5 function and targets in immune cells.

Immune cell type	Cytokine/ receptor	Substrate receptor	Substrate	Affected pathway	Result of Cul5-deficiency	Ref
HSC	IL-3	LRRC41	STAT5?	JAK2/ STAT5	Increased HSPC proliferation; Myeloid/megakaryocyte biased hematopoiesis; splenomegaly	(71)
CD41^+^ HSC	IL-3			JAK/ STAT	Increased megakaryocyte biased HSC numbers	(72)
Macrophage	IL-4				Protection against EAE; Increased M2 polarization and Arginase 1 production	
Macrophage	TLR4	–	TRAF6	NF-κB/ MAPK	Improved survival and reduced pro-inflammatory cytokine production during LPS challenge	(73)
Alveolar Macrophage	TSLP	SOCS3	OGT	IFN-β	Decreased severity of virus-induced asthma	(74)
CD4^+^ T Cell	IL-4	CIS	JAK1	JAK1/ STAT6	Increased severity of HDM-induced allergic asthma; Increased T_H_2 differentiation	(75)
CD8^+^ T Cell	IL-2/TCR	PCMTD2	IL-2B/ CD3ζ	JAK/STAT	Increased tumor cytotoxicity	(76)
NK Cell	IL-15	CIS	IL-15RB		Increased tumor cytotoxicity	(77)

### SOCS proteins in immune cell development

The process of hematopoiesis – the differentiation of immune cells from a hematopoietic stem cell into a mature effector cell – requires tight regulation to maintain a stem cell reserve while simultaneously ensuring appropriate numbers of lineage-balanced progeny. SOCS1, SOCS2, SOCS3 and CIS inhibit JAK/STAT signaling downstream of multiple cytokines required for hematopoiesis including EPO, TPO, IL-3, IL-6, G-CSF, SCF and GM-CSF (78–83). Genetic engineering studies in which SOCS proteins were deleted in one or more immune cell population helped to delineate SOCS protein function *in vivo*. Deletion of SOCS3 in mice very early in hematopoiesis resulted in neutrophilia due to prolonged STAT3 signaling downstream of G-CSF stimulation (51). Mice lacking both SOCS1 and SOCS3 in hematopoietic cells developed accelerated inflammation that was more severe than in SOCS3 deletion alone, suggesting that they have non-redundant functions to regulate cytokine signaling (82). In contrast, hematopoiesis in mice lacking SOCS2 was normal at steady-state, but following myeloablation with 5-fluorouracil, they developed excessive myeloproliferation leading to stem cell exhaustion (81). SOCS3 also plays a role in B cell development by regulating CXCL12-mediated ubiquitination of FAK to control localization in the bone marrow niche (84).

### Cul5 in immune cell development

The extent to which the functions of SOCS proteins described above are reliant on their interaction with Cul5 are not well understood. There have been limited studies on the function of Cul5 specifically in immune cell development. Two groups recently utilized a hematopoietic conditional knockout (Cul5^fl/fl^ Vav-Cre) to understand the role of Cul5 in hematopoietic stem cell (HSC) function and differentiation. One group found that Cul5 limits the production of megakaryocyte-biased HSCs (CD41^+^ HCSs) in an IL-3R dependent, and TPO and IFN-independent manner (72). Our lab demonstrated that Cul5 limits pSTAT5 downstream of IL-3 signaling in HSCs to maintain lineage-balanced, steady-state hematopoiesis. We also identified several substrate receptors outside of the SOCS family that bound Cul5 and accumulated in Cul5-deficient HSPCs, including LRRC41, WSB1, and PCMTD2 (71). Neither loss of SOCS1, SOCS2 or SOCS3 (20, 81, 82, 85) recapitulates the phenotype displayed by loss of Cul5 in either of these studies, demonstrating that Cul5 works with other substrate receptors. More research needs to be done to elucidate the composition and mechanism of CLR5 complexes that regulate function and differentiation of HSCs and progenitors.

**Figure 2:**
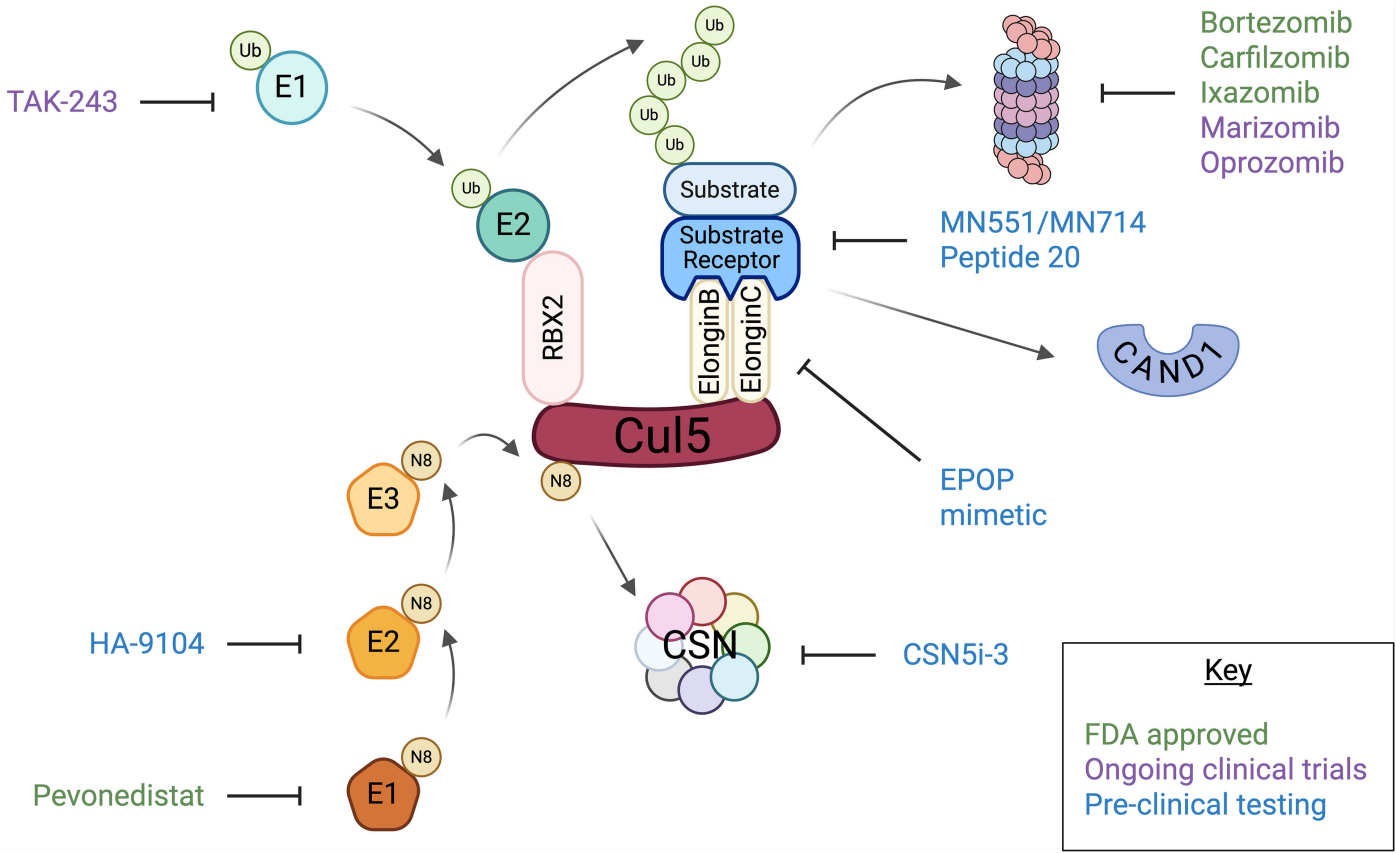
Pharmacological Targets of the UPS-CRL5 Axis. Pathways in the regulation of the CRL5 complex and drugs designed to inhibit those components. Drugs in green are FDA approved, drugs in purple are undergoing clinical trials, and drugs in blue are being investigated in pre-clinical studies. The substrate receptor binds its target substrate, then binds to Elongin B and C on Cul5 to form a CRL5 complex. Neddylation enzymes (orange pentagons) activate CRL5 by the transfer of Nedd8 to Cul5. Ubiquitin-activating and ubiquitin-conjugating enzymes (light green circles) transfer ubiquitin to RBX2, which is then transferred to the substrate. The multi-ubiquitinated substrate is transferred to the proteasome for degradation. The COP9 signalosome (CSN) removes Nedd8 from Cul5, inactivating CRL5. CAND1 removes the substrate receptor from CRL5 to allow other substrate receptors to bind.

### SOCS protein regulation of innate immunity

The innate immune system is the first line of defense against pathogens. Innate immune cells act rapidly and indiscriminately to promote pro-inflammatory pathways that sequester and neutralize the intruder. However, it is equally important to inactivate cells once the pathogen has been eliminated. CRL5 and SOCS proteins control both sides of this effort to mount an appropriate response while limiting host damage.

The innate immune response is marked by the coordination of several cell types that sense a threat, activate and recruit other cells, neutralize the pathogen and engage the adaptive immune system to mount a targeted assault and generate memory to prevent future reinfection. SOCS proteins regulate several processes in this reaction to mediate the balance of proliferation, differentiation/polarization and cytokine production in various innate cells. Dendritic cells rely on CIS, SOCS1 and SOCS2 to generate inflammatory responses but also to limit autoimmunity and maintain self-tolerance (39, 44–46). SOCS1, SOCS2 and SOCS3 are essential for the balance of pro vs anti-inflammatory polarization of macrophages (41–43, 47, 50) to control the spectrum between pathogen clearance and tissue repair. To limit excessive effector function, CIS and SOCS2 downregulate JAK1 and JAK2 downstream of IL-15 signaling to limit development, proliferation, IFNγ production and cytotoxic activity of NK cells (38, 48).

### SOCS protein regulation of adaptive immunity

The ability to recognize previously encountered pathogens and mount a specific response lies with the adaptive immune system. In addition to controlling the amplitude and duration of the innate response, SOCS proteins also regulate the coordination of the innate and adaptive immune systems, as well as the function of B and T cells that facilitate immune memory.

SOCS proteins play many roles in T cells, regulating T cell development as well as CD4^+^, CD8^+^ and T_reg_ function. SOCS1 limits IL-7 and IL-15 signaling to mediate T cell development (57–60). CIS restricts TCR dependent CD4^+^ and CD8 T^+^ cell functions including proliferation and cytokine production (53, 54). SOCS3 also impacts T cell proliferation, but through IL-2 and IL-27 regulation (68, 69). SOCS1 and SOCS3 both regulate CD4^+^ T cell differentiation into T_H_1, T_H_2 and T_H_17 cells (55, 56, 64–67). SOCS1 regulates T_reg_ function both intrinsically and through cytokine modulation in dendritic cells (61, 62). SOCS3 also controls cytokine production in NKT cells and IL-6 signaling in plasma cells, effecting germinal center function and formation (63, 70).

### Cul5 regulation of innate immunity

The role of SOCS proteins in innate immune cells has been widely studied, but the function of Cul5 is not as well understood. In recent years, there has been growing evidence that Cul5 also regulates inflammatory processes. In macrophages, Cul5 ubiquitinates TRAF6, an E3 ubiquitin ligase, to promote NF-κB activation and neutrophil recruitment in the lung in response to LPS stimulation (73, 86). During experimental autoimmune encephalomyelitis (EAE), Cul5 limits anti-inflammatory polarization and IL-4 dependent Arginase 1 production in CNS infiltrating macrophages (87). Treatment of LPS-treated mice with mitoxantrone, which inhibits NAE function and subsequently Cul5-mediated ubiquitination, resulted in decreased TAK1 function, IKK and MAPK phosphorylation, and NF-κB activation, leading to decreased pro-inflammatory cytokines and lung inflammation (88). In a model of viral-induced asthma, Cul5 ubiquitinated O-GlcNAc transferase in alveolar macrophages to inhibit IFN-β production (74). In NK cells, Cul5 and CISH facilitated the degradation of IL-15R to limit cytotoxic functions (77).

Taken together, these findings demonstrate that therapeutics designed to target SOCS proteins or Cul5 may hold value in the clinic. Modulating CRL5 functions may be valuable to enhance the innate immune response, such as during a prolonged infection. Conversely, overactive innate immune conditions such as allergy may also benefit from treatments that target CRL5 proteins.

### Cul5 regulation of adaptive immunity

Our lab identified Cul5 as the most differentially neddylated E3 in activated versus resting CD4^+^ T cells (89). Further investigation revealed that Cul5 limits T_H_2 differentiation by working with CIS to degrade JAK1 downstream of IL-4 receptor signaling (75). Cul5 also plays a role in CD8^+^ T cells by inhibiting T cell receptor and IL-2 signaling, limiting anti-tumor activity in mouse models (76).

With their roles in CD4+, CD8+, and regulatory T cells, SOCS proteins and Cul5 might serve as attractive therapeutic targets for conditions such as T cell lymphomas and leukemias, autoimmune disorders, and immunodeficiencies. These therapies could also be useful candidates for preventing transplant rejection, graft vs host disease or clearing latent viruses.

### Comparing Cul5 biology in mice and humans

The majority of the work in the studies listed in **Table 3** was performed in mouse cells and *in vivo* models, leaving open the possibility that these functions of Cul5 are not the same in humans. The sequence of the N-terminal of Cul5, where adaptor proteins bind with substrate receptors, is highly conserved between mice and humans (90). This suggests that the structure and function are perhaps also similar. Despite the strong phenotypes of Cul5 deficiency in various immune cells in mice, little evidence has linked mutations of Cul5 in humans to myeloproliferative neoplasms or asthma. It may be that there are redundancies in Cul5 function in humans that protect from developing these conditions, or that correlations in variants in the Cul5 gene and outcomes in various diseases outside of cancer and HIV have just yet to be uncovered. Two papers that aimed to improve tumor cytotoxicity of CAR-T and -NK cells found consistency in Cul5 mediated cytokine regulation between mouse and primary human CD8^+^ T cells and NK cells (77, 91). While more work is needed to confirm that these findings from mouse studies are indeed pertinent to human health, these pieces of evidence are a promising start to linking our knowledge in mice with applicability in therapeutic design. The sections below will discuss the current knowledge about Cul5 function in human biology.

## Cul5 in human disease

### Cul5 in viral infection

Multiple viruses that infect humans can hijack Cul5 function to degrade host anti-viral proteins, ensuring their own survival. During human immunodeficiency virus (HIV) infection, the virus hijacks the CRL5 complex machinery using its own substrate receptor, Vif. The Vif-CRL5 complex degrades host anti-viral proteins, called APOBECs, that prevent viral replication (92, 93). Splice variants in Cul5 have been shown to affect CD4^+^ T cell loss in patients with HIV (94), suggesting that various Cul5 haplotypes may impart functional differences in viral pathogenesis. Cul5 also plays a crucial role in protection from SARS-CoV-2 infection. Cul5 forms a complex with heat shock protein 90 alpha (HSP90α), and translocase of the outer mitochondrial membrane 70 (TOM70) to degrade ORF9b. ORF9b is an accessory protein made by the SARS-CoV-2 to evade the host immune response by inhibiting the IFN and NF-κB (95). Cul5 also has known roles in other human and animal viruses, including human adenovirus type 5 (Ad5), Kaposi’s sarcoma-associated herpesvirus (KSHV), and Epstein-Barr Virus (EBV) (22). Cul5 inhibitors may therefore be useful as antiviral drugs through several mechanisms. First, limiting the capacity of viruses to hijack the CRL5 complex for replication could result in decreased viral load. Furthermore, inhibiting Cul5 function may enhance CD8^+^ T cells’ ability to directly eliminate virus-infected cells.

### Cul5 in cancer

Differential expression of Cul5 and its correlation with patient prognosis varies widely for various cancers. Cul5 downregulation has been implicated as a marker of poor prognosis in patients with uterine cancer (96), lung cancer (97, 98), kidney cancer (99), while upregulation of Cul5 in breast cancer (100) is associated with better prognosis.

Cul5 has roles in many solid tumors, including lung, breast, ovarian, and pancreatic cancers (22, 97, 98, 100, 101). In blood cancers, Cul5 is downregulated in a subset of B-cell chronic lymphocytic leukemia (102). In cancer cell lines, Cul5 uses ASB7 as a substrate receptor to negatively regulate H3K9me3 by promoting degradation of histone methyltransferase SUV39H1. During cell division, ASB7 is phosphorylated by cyclin-dependent kinase 1 (CDK1), inhibiting its ability to degrade SUV39H1 and the restoration of H3K9me3 (103). Additionally, mice with UBE2F hematopoietic knockout had decreased colon adenocarcinoma tumor burdens compared to WT mice (77).

Cul5 also has interactions with micro-RNAs (miRNA) in cancer cells. miRNAs are small non-coding RNAs that have roles in gene expression, which can have both tumor suppressor or oncogenic functions, and are also being studied as potential targets for cancer therapies (104, 105). In hepatocarcinoma cell lines, Cul5 expression is promoted and suppressed by different miRNAs. miR-7 binds to 3’UTR-region of Cul5 to promote its expression, inducing cell-cycle arrest and reduced colony formation (106). In Hepatitis B viral infection-induced hepatocarcinoma cells, miR-145 expression negatively correlated with Cul5 expression and cell growth (107). In uterine cancer cell lines, miR-182 suppresses Cul5 expression to promote cellular proliferation (96).

The complex nature of the function of Cul5 in various types of cancers, including the mixed correlations with patient prognosis requires careful and specific research, and likely patient specific testing, to develop effective inhibitors or activators of Cul5 and CRL5 complexes.

## Clinical utility of Cul5 manipulation

Research across many clinical disciplines has implicated Cul5 function with patient outcomes. While the link between Cul5 function and human disease has not been sufficiently investigated, there is potential for clinical utility by inhibiting or promoting Cul5 activity. On one hand, promoting Cul5 function may be useful in scenarios where the immune response is disproportionate or prolonged and causing host damage. On the other, inhibiting Cul5 might be appropriate when a stronger immune response is desired, such as in patients with cancer. Outlined below are current pharmacological interventions that may benefit from insight into Cul5 function and possible therapeutics that could target Cul5 to improve patient outcomes.

### Cul5 downregulation for immune CARs

The effort of immune cells to promote self-tolerance and limit autoimmunity often comes at a detriment to their ability to perform cytotoxic functions. There is evidence that editing of Cul5 and other CRL5 components may confer cancer cell killing advantages to immune CAR cells for cancer patients. Deletion of Cul5 from CD19 CAR-T cells led to increased lymphoma cell killing in mice *in vivo* (91). Deletion of ARIH2, CUL5, CISH, UBE2F, and RNF7, components of the CRL5 complex, in CAR-NK cells led to enhanced IL-15 receptor signaling and increased cytotoxicity of a B cell cancer cell line *in vitro* (77). Although editing of CRL5 proteins for CAR therapies is still in early days, the established role of SOCS proteins and Cul5 on effector functions suggests promising targets to improve CAR efficacy.

### JAK/STAT inhibition

Dysregulation of JAK/STAT signaling can lead to malignant transformation, including the development of myeloproliferative neoplasms, acute myeloid leukemia (108–111) and T-cell acute lymphoblastic leukemia (112). Pharmacological JAK inhibitors are widely used as treatments for various autoimmune disorders, ranging from rheumatoid arthritis to myeloproliferative neoplasms (113). While these inhibitors can be efficacious, there are a few major drawbacks that mitigate their utility. Most notably, it is difficult to design a drug that will specifically target one JAK protein. Since JAKs form heterodimers, it is likely that a drug that inhibits one JAK protein will have cross-reactivity with other JAK proteins (114). This causes off target effects on multiple pathways and can lead to unintended and persistent changes even after drug clearance (115). Global inhibition of JAKs can lead to susceptibility to infection due to immunosuppression. There is also high risk for anemia, thrombocytopenia and neutropenia due to the effect of JAK inhibition on EPO, TPO and GM-CSF signaling. “Ruxolitinib discontinuation syndrome” can be caused by a rebound in high levels of cytokines that can result in symptom relapse and splenomegaly. Moreover, JAKs can activate multiple downstream pathways other than STATs, even further reducing the specificity of these drugs (116).

STAT5 is a promising clinical target due to its function in regulating proliferation of cancer stem cells (117–119). Small molecule inhibitors of STAT5 are being developed for treatment of myeloproliferative disorders and leukemias (120, 121), as well as solid cancers (122). But like JAK inhibitors, global inhibition of STAT5 may result in off target effects, due to the varied functions and targets of STAT5 in different cell types (123). Like JAK proteins, STATs form heterodimers. Therefore, targeting STAT proteins for inhibition leads to the same problems in specificity as with JAK2 inhibitors. This creates clinical utility for finding cell type or pathway specific proteins that could be pharmacologically targeted to limit off-target effects.

Cul5 and CRL5 binding partners are well known for their ability to regulate JAK/STAT signaling. But unlike global JAK or STAT inhibitors, drugs targeting specific components of CLR5 may allow for more directed effects due to the nuances of CLR5 protein expression and function in various cell types and in different disease environments.

### PROTACS

As mentioned earlier, targeted protein degradation or inhibition of protein degradation, using drugs like lenalinomide and bortezomib, respectively, have been used effectively for chemotherapeutic intervention (124, 125). Attempts are underway to harness this technology using precision medicine. There is particular interest in designing proteolysis targeting chimeras (PROTACS) that utilize engineered E3 ubiquitin ligases for targeted degradation of proteins previously considered “undruggable” (126). Even when proteins are targetable by small molecules, patients often have low response rates or develop resistance to them (127). As described in detail above, these small molecule inhibitors often have very low specificity and the potential for many off-target effects. PROTACs can be designed to be activated only in the presence of a specific stimulus. Some groups are building PROTACs that require simulation by two signals at the same time, deemed “Dual-Action-Only PROTACs,” in order to degrade their targets. This development reduces the likelihood of non-specific or unwanted degradation events and may reduce side effects and improve efficacy (128).

Few E3s are currently being utilized as PROTACs, despite the human genome encoding over 600 putative E3 enzymes. One successful PROTAC with Cul5 substrate receptors ASB1 and SOCS2 was designed to target a modified GFP substrate (129, 130), demonstrating that Cul5-based PROTACs can be successfully utilized to target proteins of interest. There are currently 10 clinical trials for PROTACs and many more are in development for hematological diseases, including myeloproliferative neoplasm, and other cancers (131, 132). Better understanding how Cul5 functions in various cell types and in response to different stimuli will aid in the design of new PROTACs.

## Pharmacological CLR5 and proteasome targets

Several small molecule inhibitors of CRL5 components or the proteasome are being developed and tested for clinical use (**Figure 2**).

### Proteasome inhibitors

The canonical function of CRL5 is to ubiquitinate target proteins which marks them for degradation in the proteasome. Proteasome inhibitors block the function of the proteasome, resulting in the accumulation of misfolded and excess proteins that build up and become toxic to the cell, leading to cell cycle arrest and apoptosis. Bortezomib is a proteasome inhibitor that reversibly binds to a subunit of the 26S proteasome. It is currently FDA approved for multiple myeloma and is also used to treat other lymphomas (133). Although it is effective in cancer cell killing, patients often experience side-effects such as peripheral neuropathy, gastrointestinal symptoms, and renal toxicity, as well as reduced red blood cells, platelets and neutrophils (134, 135). There is also evidence that bortezomib use can lead to global impairment of the immune system, potentially effecting T cells, B cells, NK cells, and dendritic cells (DCs) (136). Some patients also develop resistance to bortezomib treatment through multiple mechanisms that make the therapy less efficacious (133). Several next-generation proteasome inhibitors, including carfilzomib, ixazomib, marizomib, and oprozomib are being developed to improve on the limitations of bortezomib (137). While there are some promising results with proteasome inhibitors for treatment of multiple myeloma, more research is required to determine the subsets of patients that will benefit most from these therapies and the ideal formulation and dosing schedules to develop effective combination treatment regimens.

### Neddylation inhibitors

In order ubiquitinate target proteins, CRL5 must be activated be a post-translational modification known as neddylation. The process of neddylation has known roles in various biological functions including DNA damage response, cell cycle, and mitochondrial function (138). Dysregulation of the neddylation pathway has been linked to several diseases, including cardiac diseases and neurodegenerative diseases as well as many types of cancer (27). Neddylation inhibitors are currently being tested for solid and hematological cancers, especially acute myeloid leukemia (AML). The currently most widely tested Nedd8 inhibitor is pevonedistat, also known as MLN4924. Pevonedistat inhibits the function of the NEDD8-Activating Enzyme (NAE), preventing the activation of proteins that rely on neddylation, such as CRL5. While this class of drugs seems to have limited utility alone, they may sensitize cancers cells to other therapies and improve their efficacy in combination. However, despite promising results in Phase I and II clinical trials, a Phase III trial for AML did not reach statistical significance in event-free survival of patients treated with pevonedistat and azacitidine versus azacitidine alone (139). *In vitro* research suggests that cancer cells are able to develop resistance to pevonedistat through selection of mutations in NAE subunit UBA3 (140, 141).

E2 and E3 neddylation inhibitors are being researched, with the hope of limiting the liver toxicity and off-target effects seen with the NAE inhibitors currently being observed in clinical trials (142, 143). One group generated a small molecule inhibitor of the E2 neddylation enzyme UBE2F, HA-9104, that shows efficacy in inducing apoptosis and radiosensitivity of lung cancer cells *in vivo* and *in vivo* xenograft models (144).

### E1 ubiquitin-activating and E2 ubiquitin-conjugating enzyme inhibitors

Ubiquitination is a stepwise process that requires several different enzymes to target a specific protein for degradation. An E1 ubiquitin-activating enzyme transfers ubquitin to an E2 ubiquitin-conjugating enzyme, which then works with an E3 ubiquitin ligase, like CRL5, to transfers ubiquitin to the target protein. The human genome codes for two E1 ubiquitin-activating enzymes and around 40 E2 ubiquitin-conjugating enzymes which facilitate the transfer of ubiquitin to E3 ubiquitin ligases to direct ubiquitination of specific protein substrates. Dysregulation of UBA1, the more highly expressed of the two E1s, has been implicated in lung cancer, neurological diseases, and a newly discovered autoimmune disorder called VEXAS syndrome (145–148). A UBA1 inhibitor, TAK-243, is currently being tested in a Phase I clinical trial for acute AML and myelodysplastic syndromes. It has also shown pre-clinical efficacy in adrenocortical carcinoma, and several xenograft models (149, 150).

E2s have been implicated in numerous solid cancers including breast, prostate, lung, ovarian and liver cancers (151). Several small molecule inhibitors for specific E2s and for multiple E2s have been designed. Treatment with an RNA interference therapy was designed to target USE1, which has known roles in lung cancer, showed antitumor effects *in vitro* and in a tumor xenograft model (152). A small molecule inhibitor of Ubc13-Uev1A demonstrated to inhibit NF-kB signaling and cell survival in B-cell lymphoma cell lines (153). An anti-inflammatory compound, BAY 11-7085, which inhibits Ubc13 and UbcH7, also demonstrated antitumor effects in another B-cell lymphoma cell line (154). An allosteric inhibitor of Cdc34, which prevents the transfer of ubiquitin to p27^Kip1^, the substrate of SCF^Skp2^, and inhibited proliferation of prostate and colorectal cancer cell lines (155). One group has developed inhibitors of various E2 families, including F box proteins, and UBe2D and Ube2V members (156, 157). To our knowledge, no inhibitors of the main E2s that bind RBX2-Cul5, UBE2C and UBE2S, have been described.

### COP9 signalosome inhibitors

The COP9 Signalosome (CSN) inhibits CRL5 function by deneddylating Cul5, inactivating the complex and leading to the accumulation of CRL5 substrate receptors and substrates. CSN is composed of eight subunits, CSN1-CSN8, including CSN5, which is the catalytic subunit of the complex (158). Dysregulation of CSN subunits has been discovered in several types of cancers as well as cardiovascular diseases (159, 160). An inhibitor of CSN5, CSN5i-3, was found to inhibit growth of prostate cancer cells, breast cancer cells, and lymphoma cells *in vitro* and *in vivo* (161–163). CSN5i-3 was also shown to have synergistic effects with other cancer therapies, including PARP inhibitors and ATR inhibitors (161, 164).

### SOCS mimetics and inhibitors

As mentioned earlier, SOCS proteins can inhibit the JAK/STAT pathway in a few different ways. Among those is a feature exclusively on SOCS1 and SOCS3 called a kinase inhibitory region (KIR) that binds directly to JAKs without the need for a complete CLR5 or other E3 complex, preventing them from phosphorylating STATs. Drugs that mimic the KIR function of SOCS proteins are being explored as alternatives to JAK/STAT inhibitors (165). Cell penetrating SOCS1 and SOCS3 mimetic peptides have been synthesized and are being tested for autoimmune diseases, such as EAE and uveitis, and cancers, such as prostate cancer (166–172). Alternatively, there is an effort to develop drugs that inhibit the function of SH2 domain of SOCS proteins, like SOCS1 and SOCS2, to impair their interaction with their substrates (173, 174). Other SOCS proteins have also been implicated in human diseases and are promising targets for drug design (175).

#### Elongin B and C inhibitors

One group synthesized a peptide inhibitor of the Elongin B and C BC-box, preventing its interaction with CLR5 complexes and other binding partners. Treatment of several cancer lines with this drug impeded cell growth and induced apoptosis (176).

#### Cullin inhibitors

Promising small molecule inhibitors of Cul3 and Cul4 have been described. DI-591 selectively inhibits the neddylation of Cul3 and has shown to alleviate acetaminophen-induced liver damage in mice (177). Another group identified two small molecule inhibitors of CRL4 that exhibited antitumor effects in xenograft mouse models (178). To our knowledge, no inhibitors of Cul5 have been described. The similar, but distinct, structures of the Cullin family proteins highlight the feasibility of the design/discovery of specific inhibitors for all Cullins.

### Selecting an Appropriate CRL5 Modulating Therapy

Identifying efficacious CLR5 modulating therapies requires knowledge of the function of Cul5 and its binding partners in specific immune contexts. It is imperative to know the relationship between Cul5 and its presumed substrate receptors to select which component of the CLR5-UPS axis should be targeted for the desired therapeutic effect. For instance, SOCS3 is known to interact with Cul5 in myeloid cells. But the effects of myeloid specific SOCS3 or Cul5 deletion during EAE have opposite effects. Loss of SOCS3 results in an atypical form of the disease with exacerbated inflammation and pro-inflammatory cytokine release (179). However, Cul5 loss results in decreased CNS T cell infiltration and increase in anti-inflammatory macrophage polarization that protections mice from developing EAE (87). Therefore, using a SOCS3 mimetic may be useful for decreasing T_H_1 and T_H_17 CNS infiltration, while a direct inhibitor of Cul5 function may limit further inflammation and facilitate CNS tissue repair.

More importantly than efficacy of treatment, careful selection of a Cul5 modulating therapy is required for patient safety. As mentioned earlier, Cul5 has important roles as both a tumor suppressor and oncogene in various types of cancers. The potential for increased cancer risk with global inhibition or activation of Cul5 would need to be studied before clinical studies can move forward.

## Conclusion

The main function of Cul5 and the CRL5 complex is to target specific protein substrates for ubiquitination and subsequent proteasomal degradation, thereby limiting signaling intermediates. Through this process, CRL5 regulates a myriad of biological activities, from cell cycle and proliferation to migration and differentiation. The role of Cul5 in HIV and solid cancers has been studied extensively, but its roles in immune cell function are largely assumed due to its interaction with SOCS proteins. SOCS proteins canonically regulate JAK/STAT signaling by working with E3 complexes to degrade cytokine receptors or JAK, by blocking the pSTAT5 docking site on cytokine receptors, or by inhibiting JAK2 through a kinase inhibitory region (31). While small molecule inhibitors of Cul5 have yet to be successfully designed, several small molecule inhibitors of other CRL5 components or the UPS pathway are either FDA approved or are undergoing clinical and pre-clinical testing. Proteasome inhibitors and neddylation inhibitors are currently used in the clinic for the treatment of multiple myeloma and a number of other conditions. Despite their success, they have drawbacks that warrant the development of other drugs targeting UPS components and pathways. Inhibition or activation of CRL5 components may prove efficacious as anti-viral compounds, cancer treatments and immune modulators.
